# Complement Protein C1q Binds to Hyaluronic Acid in the Malignant Pleural Mesothelioma Microenvironment and Promotes Tumor Growth

**DOI:** 10.3389/fimmu.2017.01559

**Published:** 2017-11-20

**Authors:** Chiara Agostinis, Romana Vidergar, Beatrice Belmonte, Alessandro Mangogna, Leonardo Amadio, Pietro Geri, Violetta Borelli, Fabrizio Zanconati, Francesco Tedesco, Marco Confalonieri, Claudio Tripodo, Uday Kishore, Roberta Bulla

**Affiliations:** ^1^Institute for Maternal and Child Health, Istituto di Ricovero e Cura a Carattere Scientifico (IRCCS) Burlo Garofolo, Trieste, Italy; ^2^Department of Life Sciences, University of Trieste, Trieste, Italy; ^3^Department of Human Pathology, University of Palermo, Palermo, Italy; ^4^Department of Medical, Surgical and Health Science, University of Trieste, Trieste, Italy; ^5^Istituto di Ricovero e Cura a Carattere Scientifico (IRCCS), Istituto Auxologico Italiano, Milan, Italy; ^6^Biosciences, College of Health and Life Sciences, Brunel University London, Uxbridge, United Kingdom

**Keywords:** complement system, malignant pleural mesothelioma, hyaluronic acid, mesothelioma cells, C1q, cancer

## Abstract

C1q is the first recognition subcomponent of the complement classical pathway, which acts toward the clearance of pathogens and apoptotic cells. C1q is also known to modulate a range of functions of immune and non-immune cells, and has been shown to be involved in placental development and sensorial synaptic pruning. We have recently shown that C1q can promote tumor by encouraging their adhesion, migration, and proliferation in addition to angiogenesis and metastasis. In this study, we have examined the role of human C1q in the microenvironment of malignant pleural mesothelioma (MPM), a rare form of cancer commonly associated with exposure to asbestos. We found that C1q was highly expressed in all MPM histotypes, particularly in epithelioid rather than in sarcomatoid histotype. C1q avidly bound high and low molecular weight hyaluronic acid (HA) *via* its globular domain. C1q bound to HA was able to induce adhesion and proliferation of mesothelioma cells (MES) *via* enhancement of ERK1/2, SAPK/JNK, and p38 phosphorylation; however, it did not activate the complement cascade. Consistent with the modular organization of the globular domain, we demonstrated that C1q may bind to HA through ghA module, whereas it may interact with human MES through the ghC. In conclusion, C1q highly expressed in MPM binds to HA and enhances the tumor growth promoting cell adhesion and proliferation. These data can help develop novel diagnostic markers and molecular targets for MPM.

## Introduction

Malignant pleural mesothelioma (MPM) is a rare form of cancer that develops from cells of the pleural mesothelium and is most commonly associated with exposure to asbestos (1). MPM typically develops after a long latency period, which averages 30–40 years, and the average age of patients is 60 years (2). MPM is highly invasive to surrounding tissues leading to the failure of the organs underlying the serosal membranes (3). Although low in metastatic potential, metastasis in MPM are more frequent postsurgery; at the autopsy, metastatic diffusion is observed in 50% of patients (3). Microscopically, MPM shows mainly one of the three patterns: epithelioid, sarcomatoid, or biphasic (4).

MPM is an aggressive malignancy; most patients succumb within 2 years of being diagnosed (5). Treating MPM remains a challenge. There are two main treatment alternatives: palliative chemotherapy or multimodal treatment including surgical resection combined with chemotherapy or radiotherapy, or both (6). The resistance of MPM to conventional treatment and poor prognosis has renewed interest in basic research in order to understand the MPM biology fully with the aim of identifying possible new molecular therapeutic targets.

The local microenvironment, which encourages survival, growth, and invasion of cancer cells, plays a critical role in cancer development; the extracellular matrix (ECM) is an essential constituent of such microenvironment (7). Hyaluronic acid (HA), a member of the glycosaminoglycan family, is an abundant and ubiquitous component of the ECM (8). HA is a negatively charged high-molecular-weight (HMW) polysaccharide (4–800 kDa) which is made up of the repeating disaccharide (glucuronic acid and *N*-acetylglucosamine) (9). In the tumor microenvironment, HA offers a molecular 3D-scaffold for cells *via* the assembly of ECM, thus modulating stromal as well as tumor cells (10). HA, whose multiple functions are dictated by its molecular size and tissue concentration, relies on balanced biosynthetic and degradation processes. Increased HA synthesis has been associated with cancer progression and metastasis (11). In patients with MPM, large quantities of HA are found in the tumor tissue although both malignant and benign mesothelial cells have been found positive for intracytoplasmic HA (12).

The complement system also constitutes the local environment for cancer as an immune surveillance against malignant cells due to its ability to promote inflammation and causes direct cell killing (13). We focused our investigation on C1q, which is the first recognition subcomponent of the complement classical pathway. C1q is a potent link between innate and adaptive immunity by virtue of its ability to bind IgG- and IgM-containing immune complexes (14). In addition to being involved in the clearance of apoptotic cells, and thus maintenance of immune tolerance, C1q also has the ability to directly impact upon cell differentiation and proliferation, dendritic cell maturation, and synaptic pruning; functions that are not reliant on complement activation by C1q (15). Recently, involvement of C1q in pregnancy *via* its ability to modulate the endovascular (16) and interstitial invasion (17) of trophoblast cells in placenta has also been demonstrated. In addition, we have recently showed that C1q is present in several solid human tumor tissues and is involved in tumor progression (18).

The present study focused on the involvement of C1q in the proliferation and invasiveness of MPM. We found that C1q can bind to HA and acquires protumorigenic properties, leading to heightened adhesion, migration and proliferation of human mesothelioma cells (MES).

## Materials and Methods

### Reagent and Antibodies

Hyaluronic acid was a kind gift from Prof. Ivan Donati, Department of Life Sciences, University of Trieste (19). C1q was either purified from fresh human serum following the procedure as described previously (20) or bought from Sigma-Aldrich (Milan, Italy). The recombinant globular head regions of the A, B, and C chains (ghA, ghB, and ghC, respectively) were expressed as fusion proteins linked to maltose-binding protein (MBP) in *Escherichia coli* BL21 and purified, as described previously (21). Poly-l-lysine, bovine serum albumin (BSA) and all reagents were from Sigma-Aldrich. The following antibodies were used: monoclonal antibody (mAb) mouse anti-human C1q was from Quidel (Quidel Corporation, San Diego, CA, USA), sheep anti-human C1q and anti-human C4 were purchased from The Binding Site (Bergamo, Italy). Mouse Monoclonal anti-C5b-9 antibody (aE11) was from AbCam. Mouse mAb anti-human von Willebrand factor (vWF), mouse mAb anti-human CD68, rabbit anti-human C1q, and goat anti-mouse-FITC F(ab)′ were purchased from Dako (Milan, Italy). Mouse mAb anti-human CD44-PE, mouse mAb anti-human CD45-PE-, or FITC-conjugated, unrelated mouse IgG1-PE- and FITC-conjugated were from Immunotools (Friesoythe, Germany). Cy3-conjugated F(ab′)_2_ goat anti-mouse IgG, and FITC-conjugated F(ab′)_2_ goat anti-rabbit IgG. Mouse mAb anti-human Mesothelin and rabbit anti-human Calretinin were from Santa Cruz Biotechnology (DBA, Milan, Italy). Mouse monoclonal anti-human Vimentin, goat anti-mouse IgG alkaline phosphatase (AP)-conjugated, anti-rabbit IgG-AP-conjugated, and anti-goat IgG-AP-conjugated were from Sigma-Aldrich.

### Patients and Specimens

MPM patients who were diagnosed and followed up at the Department of Pneumology, University Hospital of Cattinara, Trieste, Italy, were enrolled for this study. None of the patients received chemotherapy or radiotherapy prior to sampling.

Patients (five male) with reported asbestos exposure underwent pleuroscopy for diagnosis of pleural effusion. All the procedures were performed under conscious sedation achieved by titration of intravenous midazolam and meperidine. Before the procedure, patients were placed in the lateral decubitus position with the pleural effusion uppermost and a bedside chest ultrasonography was used to determine the entry site. After the creation of the sterile field and injection of 2% lidocaine in the intercostal space in order to obtain local anesthesia, a 2-cm skin incision was made with a scalpel, then blunt dissection of the chest wall was performed using curved Kelly forceps down to the parietal pleural. Finally, a trocar was placed into the pleural space and the pleuroscope (Karl Storz GmbH, Tuttlingen, Germany) was inserted to examine parietal and visceral pleura to obtain parietal pleural specimens using dedicated forceps. At the end of the procedure, pleuroscope and trocar were removed and a chest tube was inserted trough the chest wall.

Tissue samples from patients were collected after informed consent following approval of the ethical considerations by the Institutional Board of the University Hospital of Trieste, Italy.

### Cell Isolation and Culture

Mesothelioma cells were isolated from pleural biopsy specimens. The tissue was finely minced with a cutter, incubated with a digestion solution composed of 0.5% trypsin (Sigma-Aldrich, Milan, Italy) and 50 µg/ml DNase I (Roche, Milan, Italy) in Hanks’ Balanced Salt solution with Ca^2+^Mg^2+^ 0.5 mM (Sigma-Aldrich) overnight at 4°C. Next, the enzymatic solution was replaced with collagenase type 1 (3 mg/ml) (Worthington Biochemical Corporation, DBA) diluted in Medium 199 with Hank’s salts (Euroclone Spa, Milan, Italy) for 30 min at 37°C. The digestion was blocked with 10% fetal bovine serum (FBS, GIBCO, Life Technology) and the cell suspension was filtered through a 100 µm pore filter (BD Biosciences, Italy).

The cells were seeded in a 12.5 cm^2^ flask and cultured using Roswell Park Memorial Institute (RPMI) medium 1640 with GlutaMAX (Life Technologies, Milan, Italy), 45% human endothelial cells serum-free medium (HESF, Life Technologies), 10% heat-inactivated FBS supplemented with EGF (5 ng/ml), basic FGF (10 ng/ml), and penicillin–streptomycin (Sigma-Aldrich). Fresh medium was replaced every 2–3 days. MES were used at their five to eight passages for all the *in vitro* experiments.

Met5A cells were purchased from ATCC. These cells were grown in DMEM supplemented with 10% FBS and 1% antibiotic mixture (Sigma-Aldrich) and maintained at 37°C in humidified atmosphere with 5% CO_2_.

### Pleural Effusions

Malignant pleural effusions (MPEs) were obtained from patients who underwent thoracentesis after diagnosis of MPM in order to remove the exudative liquid filling pleural space. The surgery was performed within the Department of Pathologic Anatomy of the Hospital of Monfalcone (Gorizia, Italy). MPEs were immediately stored at 4°C for a maximum of 24 h before being processed. Approximately 1 Liter of MPE was centrifuged twice at 250 *g* for 10 min to remove the cells.

### Dose-Determination of Soluble C1q

A 96-well plate (Corning Costar) was coated with sheep anti-C1q (1:6.000) diluted in carbonate/bicarbonate buffer (100 mM, pH > 9) and incubated overnight at 4°C. In order to avoid non-specific binding, the microtite wells were blocked with 2% skimmed milk (SM) in PBS and incubated for 2 h at 37°C. In the meanwhile, samples to be dose-titrated were prepared and then added to the wells in triplicate. A standard curve was prepared using a serial dilution of purified C1q (Sigma-Aldrich) from 50 to 1.56 ng/ml and the plate was incubated overnight at 4°C. Rabbit anti-human C1q (1:1,000) diluted in PBS + 0.5% SM + 0.05% Tween was incubated for 1 h at 37°C, followed by secondary probing with anti-rabbit IgG-alkaline phosphatase (AP) conjugate (1:20,000) for 30 min at 37°C. *p*-nitrophenyl phosphate (pNPP) was used as substrate, as described above, and the developed color was measured at 405 nm using the Titertek Multiskan ELISA Reader (Flow Labs).

### Immunohistochemical Analysis

Tissue samples of different MPM histotypes were fixed in 10% buffered formalin and paraffin embedded. Sections of 4 µm in thickness were fixed with xylene, 100% EtOH, and 95% EtOH and then microwaved three times in Tris–HCl/EDTA pH 9.0 buffer (Dako, Milan, Italy) for 5 min and washed in Tris-buffered saline. After neutralization of the endogenous peroxidase with H_2_O_2_ (hydrogen peroxide) for 10 min, the sections were first incubated with PBS + 2% w/v BSA + 0.4% w/v Casein for 5 min in order to block the non-specific sites, and then probed with rabbit anti-human C1q (1:500) overnight at 4°C. The bound antibodies were revealed using the Vectastain Elite ABC horseradish peroxidase (HRP) kit (Vector Laboratories, DBA, Italy). Secondary antibodies were detected by 3-amino-9-ethylcarbazole (AEC) + high sensitivity Chromogen (Dako). The sections were counterstained with hematoxylin (Dako). Slides were examined under a Leica DM 3000 optical microscope and images were collected using a Leica DFC320 digital camera (Leica Microsystems, Wetzlar, Germany).

### Alcian Blue Staining

After deparaffinizing and rehydrating, the sections were incubated with a solution of 1% Alcian blue dissolved in 3% Acetic acid, pH 2.5, for 30 min at RT. After washing in tap water for 10 min, the sections were dehydrated well in absolute alcohol and mounted. Images were acquired by the fluorescence microscope Leica DM 3000 using the Leica DFC320 camera.

### Immunofluorescence Microscopy of MES

Mesothelioma cells cultured at confluence in an eight-chamber slide (BD Falcon) were fixed with FIX&PERM kit (Invitrogen, Life Technologies) for 15 min at RT. Incubation with primary antibodies (as listed earlier) was carried out for 1 h at RT. Cells were then washed and incubated with corresponding secondary antibodies (1:300) for 45 min at RT. The nuclei were stained with DAPI (Sigma-Aldrich). The glass was mounted with the Fluorescence Mounting Medium (Dako) and covered with a cover slip. Images were acquired by the fluorescence microscope Leica DM 3000 using the Leica DFC320 camera.

### Flow Cytometry

Mesothelioma cells (5 × 10^5^) were fixed with the fixation reagent FIX&PERM kit for 15 min at RT in dark and incubated with primary antibodies for 1 h at 37°C in a thermomixer (Eppendorf) at 800 rpm. Antibodies directed against intracellular antigens were diluted in permeabilization reagent of the FIX&PERM kit, while antibodies for cell surface antigens were diluted in PBS-1% w/v BSA. Incubation with secondary antibodies anti-mouse-FITC F(ab)′ (1:50) or anti-rabbit-FITC (1:300) was performed for 30 min on ice. Cells were suspended in 1% paraformaldehyde, the fluorescence was acquired with the FACScalibur (BD Bioscience), and data processed using the software CellQuest.

### Binding of C1q to MES

Mesothelioma cells were grown to confluence in 96-well tissue culture plates and incubated directly with increasing concentrations of purified human C1q or preincubated with increasing concentrations of HA, for 1 h at room temperature. Bound C1q was revealed by ELISA using mAb anti-human C1q (10 µl/ml) and alkaline-phosphatase-conjugated secondary antibodies (Sigma-Aldrich). The color, developed using pNPP (Sigma-Aldrich; 1 mg/ml) as a substrate, was read at 405 nm using a Titertek Multiskan ELISA reader (Flow Labs, Milano, Italy).

### Coating Conditions

The microtiter wells were coated overnight at 4°C with HMW HA (50 µg/ml), C1q (20 µg/ml), ghA, ghB, ghC, or BSA (as a negative control; Sigma-Aldrich) diluted in 100 mM carbonate/bicarbonate buffer, pH 9.6. C1q was allowed to bind (25 µg/ml) to HA in PBS + 0.5% BSA, 0.7 mM CaCl_2_, and 0.7 mM MgCl_2_, overnight at 4°C.

### Adhesion Assay

1 × 10^5^ mesothelial cells or MES, labeled with the fluorescent dye FAST DiI (Molecular Probes, Invitrogen), were re-suspended in HESF (Life Technologies) containing 0.1% BSA (HESF + 0.1% BSA; Sigma), preincubated with 10 µM of ERK (#SCH772984, Selleckchem), JNK (#SP600125, SIGMA-Aldrich), or p38 (#SB203580, Selleckchem) inhibitors for 30 min at RT and then added to a 96-well plate (wells were coated as described above) for 35 min at 37°C in 5% v/v CO_2_ incubator. Then, the unbound cells were removed and the adherent cells were lysed with 10 mM Tris–HCl, pH 7.4 + 0.1% v/v SDS. The plate was read *via* Infinite200 (544 nm, emission 590 nm) (TECAN) using a calibration curve generated through an increasing number of labeled cells.

### Cell Proliferation

The cell proliferation assay was performed using Click-iT^®^ Plus EdU Proliferation Kits (ThermoFisher). 5 × 10^3^ MES were re-suspended in HESF + 0.1% BSA medium and seeded to a 96-well plate, which was earlier coated with C1q, HA or C1q + HA, as described above. Following adhesion, cells were incubated with 15 µM analog EdU nucleotide (5-ethynyl-2-deoxyuridine) for DNA incorporation during replication. Cells were then incubated for 24 h at 37°C, fixed, and set up for a marking reaction by azide Oregon-Green 488. The signal amplification step included the incubation with antibody anti-Oregon-Green conjugated with HRP that reacts with the substrate, Amplx UtraRed, and produces a bright response that beams fluorescence around red. The fluorescence was analyzed by TECAN (Tecan, Milan, Italy) in the excitation/emission range of 535/595 nm.

### Apoptosis

Mesothelioma cells grown without serum, were suspended in 0.1% w/v BSA in HEFS, and 2 × 10^4^ cells/well were seeded on precoated plates. The cells were left to adhere for 1 h at 37°C, then incubated with 500 µM of H_2_O_2_ for 6 h, before adding 5 µM of CellEvent™ Caspase-3/7 Green Detection Reagent (Life Technologies), a fluorogenic substrate for activated caspases 3 and 7.

### Migration Assay

FAST DiI-labeled (Molecular Probes, Invitrogen, 1:100) MES (2 × 10^5^ cells) were resuspended in 0.1% w/v BSA in HEFS and added to the upper chamber of a transwell system. The cells were allowed to migrate through HTS FluoroBlok systems with polycarbonate membranes of 8 µm pore size (Falcon) coated on the lower side, as described above. The plate was read using Infinite200, as described above.

### Scratch Assay

Confluent monolayers of MES (2 × 10^5^) were seeded in HESF + 0.1% BSA medium in 24-well plate. A scratch was placed in the middle of the well with a sterile 200 µl pipette tip. Subsequently, HA (50 µg/ml) or HA + C1q (20 µg/ml) were added to the wells. Cells incubated with 10% v/v FBS and as negative control MES were cultured in HESF/BSA (0.1%) medium without stimuli. Images were acquired by phase-contrast microscope (Leica).

### Phosphorylation of ERK, SAPK/JNK, and p38 in MES

Pathway analysis was performed as per the manufacturer’s instructions of the PathScan^®^ Intracellular Signaling Array Kit (Fluorescent Readout) (#7744; Cell Signaling Technology, EuroClone, Milan, Italy). Briefly, 24 h serum-starved MES (1.8 × 10^6^ cells) were left to adhere to HA, or HA-bound-C1q, as described above for the indicated periods of time at 37°C. Then, the cells were washed with ice-cold 1× PBS and lysed in 1× ice-cold Cell Lysis buffer containing a cocktail of protease inhibitors (Roche Diagnostics). The Array Blocking Buffer was added to each well and incubated for 15 min at RT. Subsequently, an equal amount of total lysate (0.8 mg/ml) was added to each well and incubated for 2 h at RT. After washing, the biotinylated detection antibody cocktail was added to each well and incubated for 1 h at RT. Streptavidin-conjugated DyLight 680 was added to each well and incubated for 30 min at RT. Fluorescence readout was acquired using the LI-COR Biosciences Infrared Odyssey imaging system (Millennium science) and data processed by the software Image studio 5.0.

### Detection of the Interaction between Human C1q (and Its Recombinant Globular Head Modules) and HA

The microtiter wells were coated overnight at 4°C with either 50 µg/ml HA of different MWs diluted in carbonate/bicarbonate buffer (100 mM, pH 9.6). The blocking step with PBS + 1% BSA (2 h at 37°C) was followed by incubation with an increasing concentration of human C1q or the recombinant globular head modules (ghA, ghB, and ghC) of human C1q in PBS-Ca^2+^Mg^2+^ (0.7 mM) containing 0.5% BSA (PBS-CaMg-0.5% BSA) overnight at 4°C. Bound C1q was detected with sheep anti-human C1q polyclonal antibodies whereas bound ghA, ghB, and ghC were detected with mouse anti-MBP (1 h at 37°C). The binding of anti-C1q was detected using anti-goat IgG-AP conjugate, whereas the anti-MBP binding was detected using anti-mouse IgG-AP conjugate for 30 min at 37°C. The phosphatase substrate, pNPP (Sigma-Aldrich) was dissolved in 0.1 M *glycine buffer* containing 1 mM MgCl_2_, 1 mM ZnCl_2_, pH 10.4 at a concentration of 1.5 mg/ml. The absorbance was measured at 405 nm with the Titertek Multiskan ELISA Reader (Flow Labs).

### Evaluation of Complement Activation

Microtitre wells in a 96-well plate were coated with HA (50 µg/ml) or IgG (10 µg/ml), as described above. Non-specific binding sites were blocked with PBS-1%BSA for 1 h at 37°C, and then incubated with 20 and 50 µg/ml C1q diluted in PBS-CaMg-0.5%BSA for 1 h at 37°C. Subsequently, the wells were incubated with pooled normal human serum (1:100) as a source of complement components in PBS-CaMg-0.5%BSA and incubated for 30 min at 37°C with gentle shaking. C9 neoantigen detection was performed using the murine mAb aE11 against C9 neoantigen (kindly provided by Prof. T. E. Mollnes, Oslo, Norway) and incubated for 1 h at 37°C. The AP-conjugated anti-goat IgG (Sigma-Aldrich) or anti-mouse IgG (Sigma-Aldrich), used as secondary antibodies, were incubated 30 min at 37°C. pNPP (Sigma) was dissolved in glycine buffer at the concentration of 1.5 mg/ml. The absorbance was measured at 405 nm with the Titertek Multiskan ELISA Reader (Flow Labs).

### Statistical Analysis

Data were analyzed using Two-way ANOVA, Tukey–Kramer test, and unpaired two-tailed Student’s *t*-test or one-way ANOVA with Bonferroni corrections. Results were expressed as mean ± SEM. Non-parametric data were assessed by Mann–Whitney *U* tests and the results were expressed as median and interquartile range. *p* values < 0.05 were considered significant. All statistical analyses were performed using Prism 6 software (GraphPad Software Inc., La Jolla, CA, USA).

## Results

### C1q Is Present in Malignant Pleural Mesothelioma Specimens

**Figure 1:**
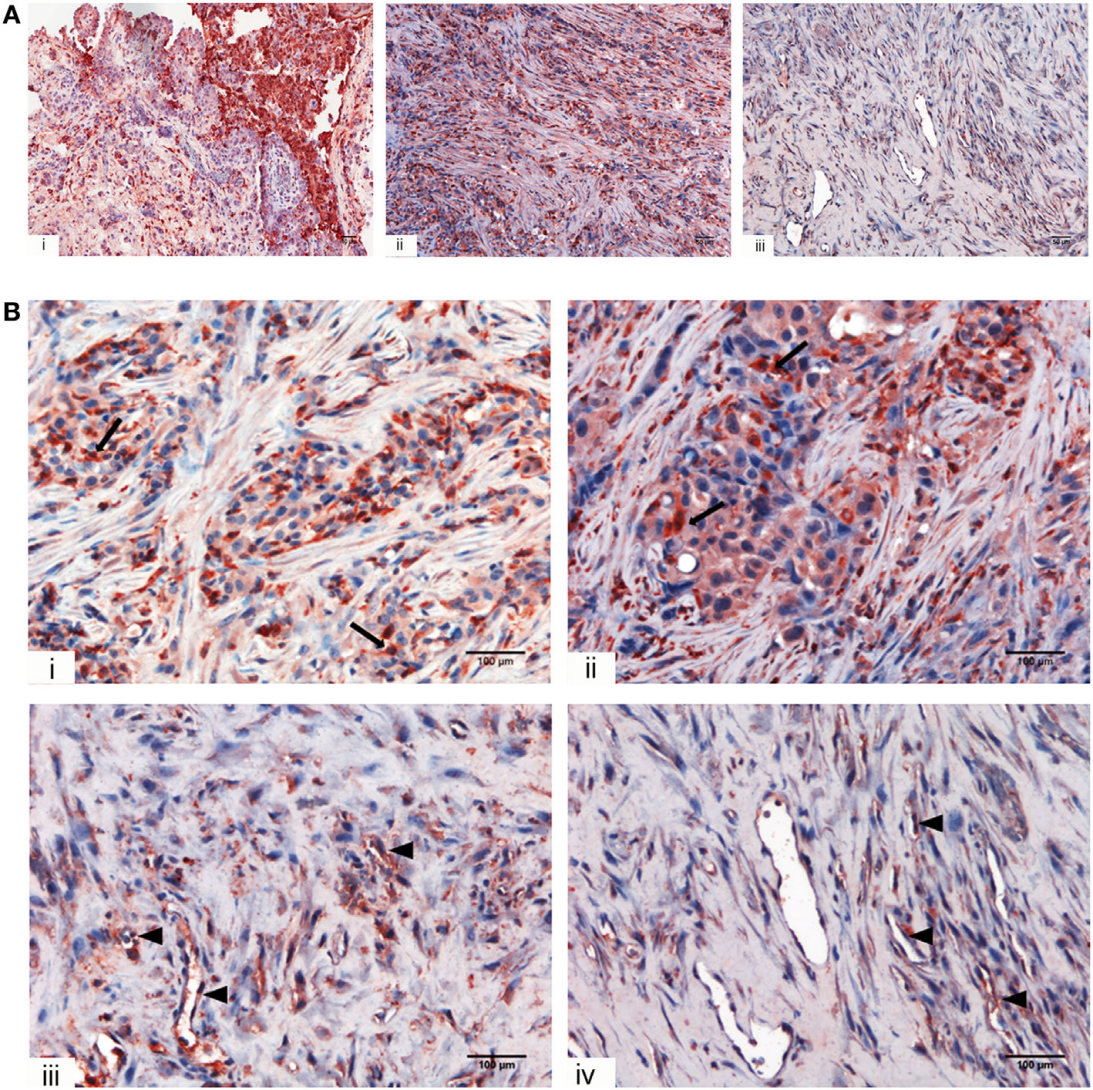
Immunohistochemical analysis for C1q in human mesothelioma. **(A)** Representative microphotographs showing expression of C1q in different malignant pleural mesothelioma (MPM) histotypes: epithelioid (i), biphasic (ii), and desmoplastic (iii). Streptavidin–biotin–peroxidase complex system with 3-amino-9-ethylcarbazole (AEC) (red) chromogen; scale bars, 50 µm. **(B)** Representative microphotographs showing the expression of C1q in tumor-associated stroma of mesothelioma. Highlighted are monocytoid cells suggestive of tumor-infiltrating myeloid elements (arrows) and neovascular endothelial cells (arrow heads). Streptavidin–biotin–peroxidase complex system with AEC (red) chromogen; scale bars, 100 µm.

We initially looked for the presence of C1q in a panel of invasive MPM specimens, including epithelioid, biphasic, and sarcomatoid (Figure 1A) histotypes. As shown in Figure 1, a strong positivity for C1q was detected in all tumor specimen types examined, particularly in epithelioid histotype.

**Figure 2:**
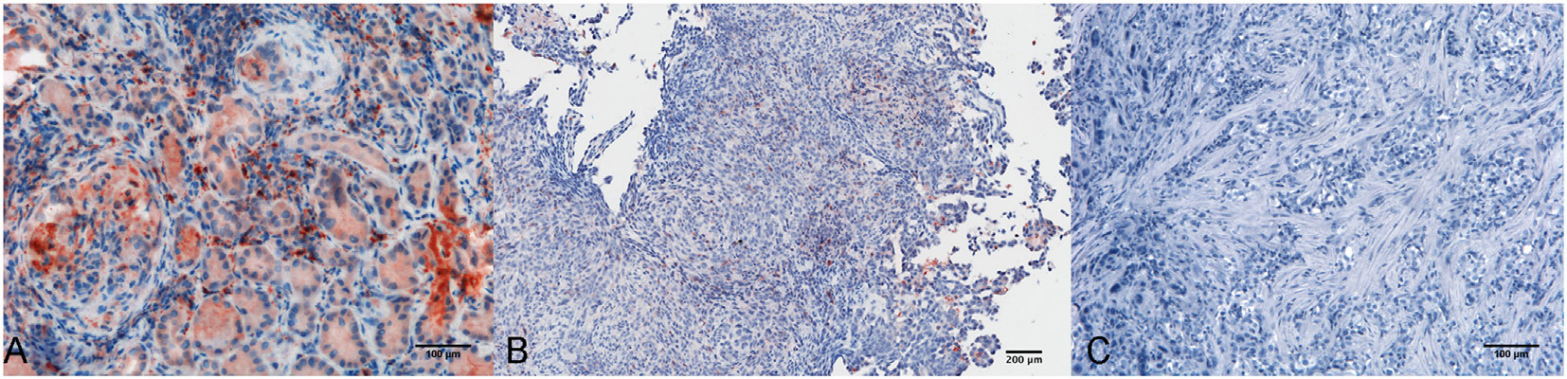
Immunohistochemical controls for C1q. Representative microphotographs showing the immunohistochemistry controls: positive control tissue, lupus nephritis **(A)**, scale bar, 100 µm; negative control tissue, pleural benign cyst **(B)**, scale bar, 200 µm; negative control reaction, biphasic mesothelioma **(C)**, scale bars, 100 µm. Streptavidin–biotin–peroxidase complex system with 3-amino-9-ethylcarbazole (AEC) (red) chromogen.

Within the mesothelioma microenvironment, C1q was mainly expressed by monocytoid cells suggestive of tumor-infiltrating myeloid elements (shown by arrows in the upper panels) and in the small vessels, as indicated by the black triangles of the lower panels in Figure 1B. C1q was also diffusely present in the tumor stroma and associated with the cell membrane of tumor cells. Immunohistochemical controls of C1q are shown in Figure 2. The presence of C1q was also detected in the three pleural exudate by a quantitative ELISA assay. We found out that the concentration of C1q was found to be about 76 µg/ml (±8 μg/ml), approximately two- or threefold lower than our control serum (about 200 µg/ml).

### Binding of C1q to HA does not Activate the Complement Classical Pathway

**Figure 3:**
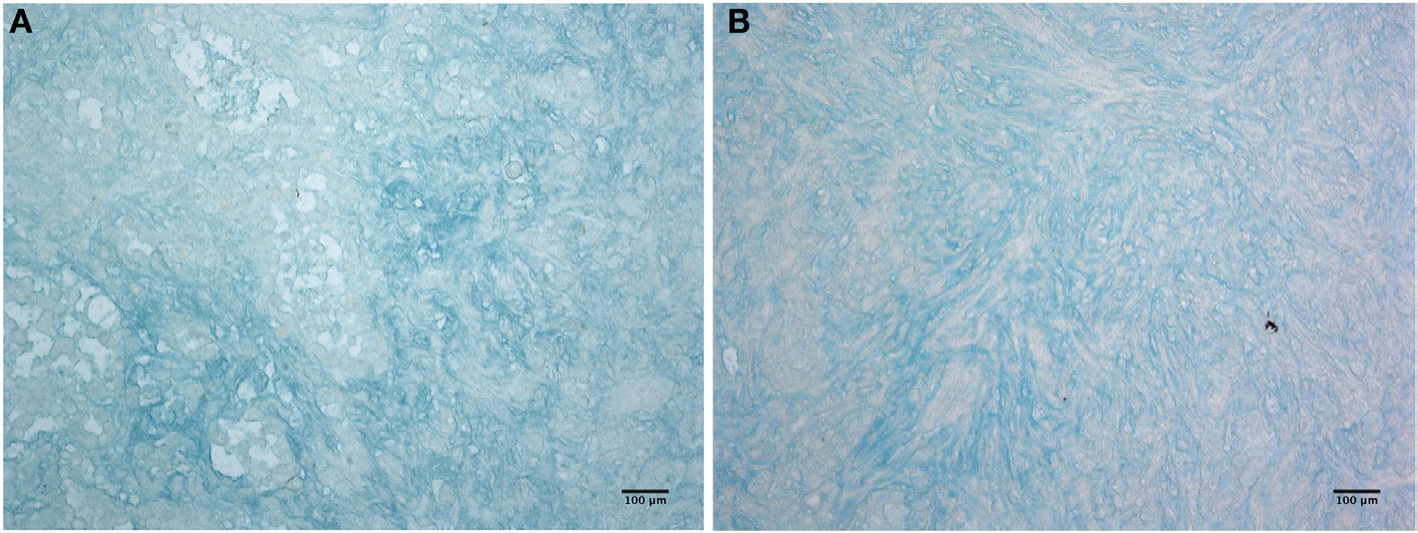
Histochemistry of hyaluronic acid on malignant pleural mesothelioma (MPM). Alcian blue staining highlights hyaluronic acid of epithelioid **(A)** and biphasic **(B)** malignant mesothelioma sections. Scale bars, 100 µm.

Having shown that C1q is abundantly present in the mesothelioma microenvironment, we investigated the ability of C1q to interact with ECM components. We focused our attention to HA which is abundantly present in mesothelioma tissue (22). The presence of HA is clearly evident in Figure 3, where we show sections obtained from epithelioid and a biphasic mesothelioma tissue stained with Alcian blue.

**Figure 4:**
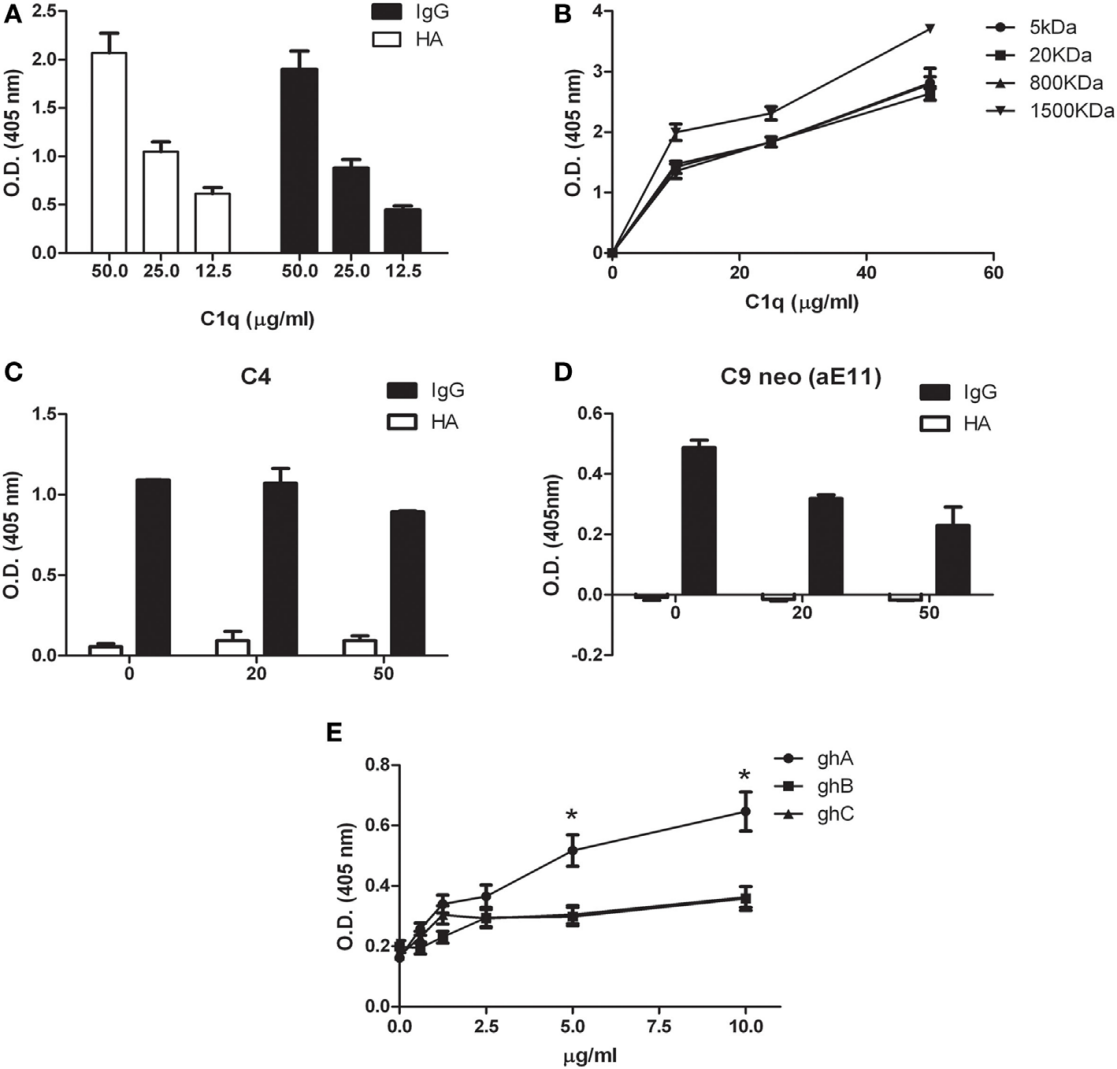
Interaction of C1q with hyaluronic acid. **(A)** C1q interaction with high-molecular-weight (HMW) hyaluronic acid (HA) and IgG (positive control) by ELISA. Microtiter wells coated with 50 µg/ml HMW HA or 10 µg/ml IgG were incubated with an increasing concentration of C1q; HA-bound C1q was revealed with anti-C1q antibodies. C1q bound HA with an affinity similar to IgG. The data are expressed as mean of three independent experiments in triplicates ± SEM. **(B)** Binding of C1q to different MW HA. Microtiter wells coated with 50 µg/ml HMW-HA (or 5, 20, 800, or 1,500 kDa HA) were incubated with increasing concentration of C1q and bound C1q was revealed with anti-C1q antibodies. No statistically significant difference was observed between the binding of C1q to low and high MW-HA was observed. The data are expressed as mean of three independent experiments carried out in triplicates ± SEM. **(C–D)** Evaluation of the ability of C1q bound to HA to activate the classical pathway of the complement system. Microtiter wells coated with 50 µg/ml HMW HA, or 10 µg/ml IgG were incubated with 20 and 50 µg/ml C1q. Pool of normal human sera (1:100) was added as a source of complement components and the deposition of C4 **(C)** and the formation of C9-neoantigen (C9 neo) **(D)** were revealed by using specific antibodies against C4 and C9 neoantigen (mAb aE11) by ELISA. The data are expressed as mean of three independent experiments carried out in triplicates ± SEM. **(E)** Dose response curve of the binding of ghA, ghB, and ghC to HMW HA. Microtiter wells were coated with 50 µg/ml HMW HA. The wells were then incubated with increasing amounts (0–10 µg/ml) of recombinant C1q globular head modules (ghA, ghB, ghC). The binding was revealed using anti-MBP antibody. The data are expressed as mean of three independent experiments done in triplicates ± SEM.

We previously demonstrated that C1q is able to bind to a range of target ligands present in the ECM; this interaction is particularly strong with HA (17). We confirmed by ELISA the ability of C1q to bind to HA in a manner similar to IgG (Figure 4A). We also analyzed the binding of C1q to HA of different molecular weights (Figure 4B). Our results indicated that there were no statistically significant differences in C1q binding to low and high MW-HA. Furthermore, we evaluated the capability of HA-bound C1q to activate the complement classical pathway, by measuring the C4 and C9 (neoantigen) (C9 neo) deposition by ELISA. As shown in Figures 4C,D, only C1q bound to IgG, and not HA-bound C1q, induced complement activation, and therefore, C4 deposition and C9-neoantigen formation. In order to localize the C1q interaction with HA, we analyzed the binding of recombinant forms of individual globular head modules (ghA, ghB, and ghC) to HA. Our results indicated that the globular head of C1q A chain (ghA) bound to HA better than ghB and ghC (Figure 4E), suggesting a differential and modular nature of the interaction between the gC1q domains and HA.

### Isolation and Characterization of Primary Tumor Cells from MPM Biopsies

**Figure 5:**
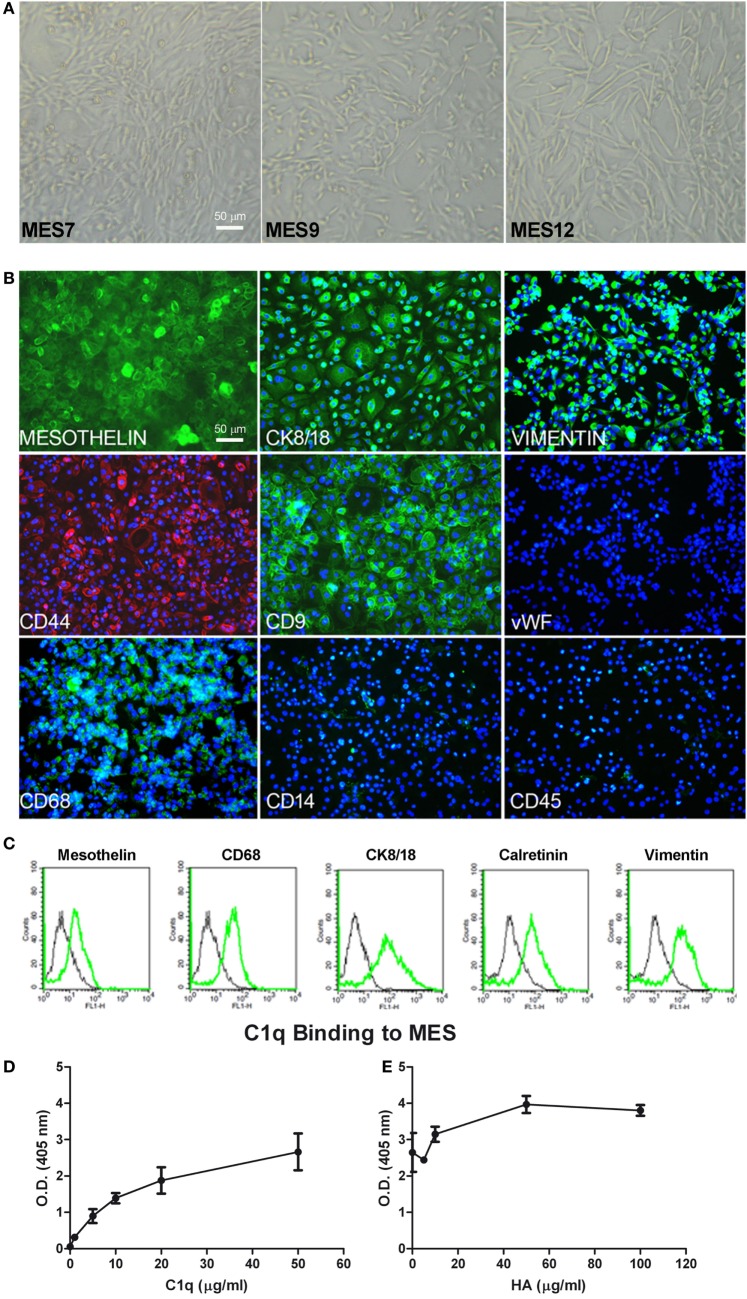
Characterization of mesothelioma cells (MES). **(A)** Morphological features of the three different populations of MES (MES7, MES9, and MES12) isolated from malignant pleural mesothelioma (MPM) biopsies. Images were acquired by phase-contrast microscope, Leica original magnification: 200×. **(B)** Mesothelial cells were characterized by immunofluorescence for the expression of mesothelin, CK 8/18, vimentin, CD9, CD68 (green), and CD44 (red). Mesothelioma cells were grown to confluence in eight-chamber culture slides. After fixation and permeabilization, the cells were stained with mAb anti-human mesothelin, CK 8/18, vimentin, CD9, von Willebrand factor (vWF), CD68, and CD14, followed by anti-mouse-FITC F(ab)′ secondary antibodies or mAb anti-human CD45 and mAb anti-human CD44-PE conjugate. Nuclei were stained blue by DAPI: original magnification 200×. **(C)** The expression of mesothelin, CD68, CK8/18, calretinin, and vimentin was confirmed by FACS. The expression of these markers (green lines) was compared with appropriate control antibodies (black lines). **(D)** Binding of C1q to MES. Tumor cells grown to confluence on 96-well tissue culture plates were incubated with increasing concentrations of purified C1q for 1 h at room temperature and the bound C1q was revealed by ELISA. **(E)** The binding of 25 µg/ml C1q to MES was detected preincubating the cells with increasing concentration of hyaluronic acid (HA). Bound C1q was revealed by ELISA as described above. The data are presented as mean ± SEM of three separate experiments.

Having established that C1q is present in the MPM microenvironment and that it can bind to HA, we sought to investigate the implication of its presence in MPM biology. Therefore, we isolated MES from a portion of the resected malignant pleura, obtained during diagnostic pleuroscopy, from five patients with epithelioid MPM. We compared the MES morphology with Met5A, a commercial, immortalized mesothelial cell line, commonly used as a model of healthy cells. MES had mainly an elongated and filamentous shape and were very heterogeneous and multishaped (Figure 5A). It is possible to notice that there are also some polygonal and more regular cells in culture, which seem to resemble the epithelial phenotype of Met5A. Generally, they had an abundant cytoplasm in which vacuoles or granules were often present, transforming themselves in signet-ring cells.

We characterized MES for the expression of typical mesothelial markers, such as mesothelin, calretinin, CK8-18, and CD44 by immunofluorescence microscopy. The cells were also positive for vimentin, a marker of mesenchymal cells (Figure 5B). MES were positive for the above-mentioned markers. To assess the purity of isolated primary cells, we performed immunophenotypical staining against the classical leukocyte antigen CD45 to avoid the presence of contaminating leukocytes. Furthermore, we also found that MES cells were negative for vWF, a common marker used to detect endothelial cells. Interestingly, all MES were positive for CD68, a mature macrophage marker, although they were negative for CD14. Some of these markers were also analyzed by FACS (Figure 5C; Table 1).

**Table 1:** Marker expression evaluated by FACS analysis on cells isolated from five different epithelioid mesothelioma tumors.

Marker	MES7	MES9	MES12	MES13	MES14
Mesothelin	+	+	+	+	+
Vimentin	++	++	+	+	+
CK 8/18	++	Nd	+	+	+
Calretinin	+	Nd	−	+	+
vWF	−	−	−	Nd	−
CD68	++	+	+	+	+
CD45	−	−	−	−	−
CD44	++	+	Nd	Nd	++

*Data refer to cells between the third and the fifth passage in culture. ++ = positivity (>50%), + = positivity (<50%), − = negativity, Nd = not done*.

IHC-positive staining for C1q in MPM tissues suggested that C1q may be produced locally. In this regard, C1q expression was evaluated at both mRNA and protein levels in healthy (Met5A) and tumor MES. We performed qPCR for three C1q chains (*C1qA, C1qB*, and *C1qC*); none were positive in five different cell populations (data not shown). Interestingly, C1q was found to bind strongly to the surface of MES (Figure 5D) and the extent of binding increased in the presence of HA being highest at the concentration of 50 µg/ml of HA (Figure 5E). To investigate the role of bound C1q on complement activation, we incubated C1q-bearing cells with AB+ human serum and analyzed the cells for the presence of C4 and C9 neoantigen on their surface. We failed to detect both complement components suggesting that binding of C1q to MES is not associated with complement activation.

### C1q Promotes MES Adhesion and Spreading

**Figure 6:**
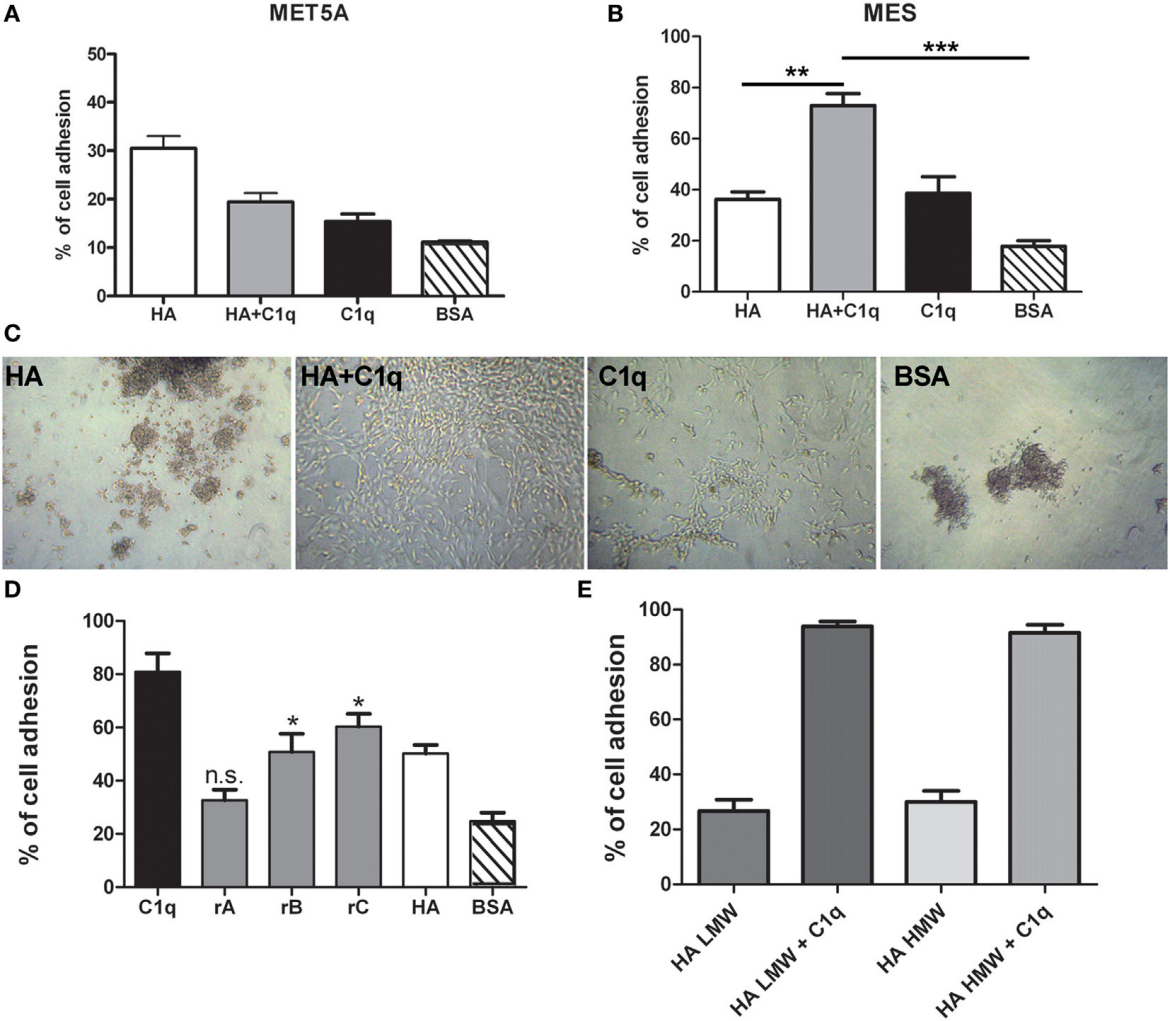
Effect of C1q on mesothelioma cells (MES) adhesion. Mesothelial cells (Met5A) **(A)** and four different populations of MES **(B)** were labeled with the fluorescent dye FAST DiI and allowed to adhere to microtiter wells precoated with hyaluronic acid (HA), C1q, HA-bound-C1q, or bovine serum albumin (BSA). The data are expressed as mean of four independent experiments done in triplicates ± SEM. Cell adhesion was measured after 35 min. Results are expressed as percent of adhesion with reference to a standard curve established using an increasing number of labeled cells. **(C)** Morphological aspect of one representative primary cell line adhered to HA, C1q, HA-bound-C1q, or BSA. Images were acquired *via* phase-contrast microscopy, original magnification: 200×. **(D)** MES cells were labeled with the fluorescent dye FAST DiI and allowed to adhere to microtiter wells precoated with C1q, ghA, ghB, ghC, high molecular weight (HMW)-HA, or BSA. The data are expressed as mean of four independent experiments carried out in triplicates ± SEM. **(E)** MES were labeled with the fluorescent dye FAST DiI and allowed to adhere to microtiter wells precoated with HMW-HA, HMW-HA + C1q, low molecular weight (LMW)-HA, or LMW-HA + C1q. The data are expressed as mean of four independent experiments carried out in triplicates ± SEM.

**Figure 7:**
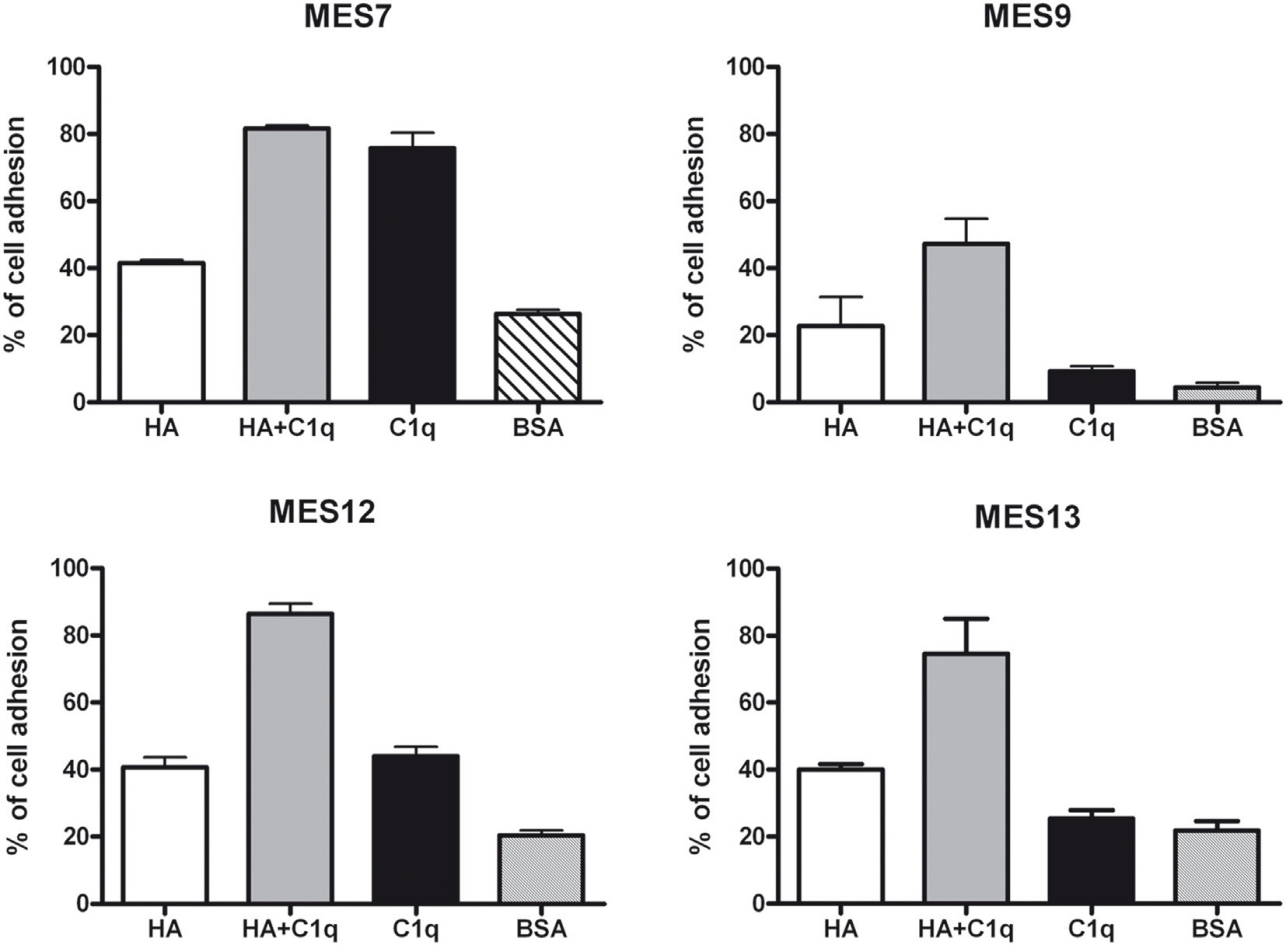
Effects of C1q on mesothelioma cells (MES) adhesion. Four different populations of MES were labeled with the fluorescent dye FAST DiI and allowed to adhere to microtiter wells precoated with hyaluronic acid (HA), C1q, HA-bound-C1q, or bovine serum albumin (BSA). The results of each independent experiment, done in triplicates ± SEM are shown in order to highlight differential behavior of these cells. Cell adhesion was measured after 35 min. The results are expressed as percent of adhesion with reference to a standard curve established using an increasing number of labeled cells.

To evaluate the ability of C1q to interact with MES, we performed a cell adhesion assay using immobilized C1q. MES (four different populations) and Met5A cells were labeled with the fluorescent probe FAST DiI and seeded on to immobilised C1q, HA or HA-bound-C1q; BSA was used as a negative control protein. Met5A cells were able to adhere to HA, and to a less extent, to C1q or HA-bound-C1q (Figure 6A). In contrast, MES showed greater adherence to HA; HA-bound-C1q was able to enhance MES adhesion considerably compared to HA alone (Figure 6B). All four MES populations showed the same behavior on HA-bound-C1q, although MES adhesion to C1q alone varied considerably between patients’ samples (Figure 7).

The analysis of the adherent MES by phase-contrast optical microscopy revealed that a high proportion of the cells seeded on to C1q or HA-bound-C1q were spread out, in contrast to the round morphology exhibited by those attached to HA or BSA (Figure 6C). In order to understand which module of the globular domain of C1q was mainly involved in MES adhesion, MES were stained with Fast DI and allowed to adhere to C1q, HA or recombinant globular heads (ghA, ghB, ghC) for 30 min. As shown in Figure 6D, the interaction of the cells with C1q seems to be mediated by ghC, and to a lesser extent, ghB. The cells did not seem to interact with ghA.

Since both HMW-HA and LMW-HA are present in the tumor microenvironment, we investigated the adhesion of MES to LMW-HA alone or in combination with C1q and we compared the results with the adhesion to HMW-HA with or without C1q. We did not observe any statistical difference between the MES adhesion to LMW or HMW-HA (Figure 6E).

### C1q Promotes MPM Tumor Progression by Favoring MES Proliferation and Migration

**Figure 8:**
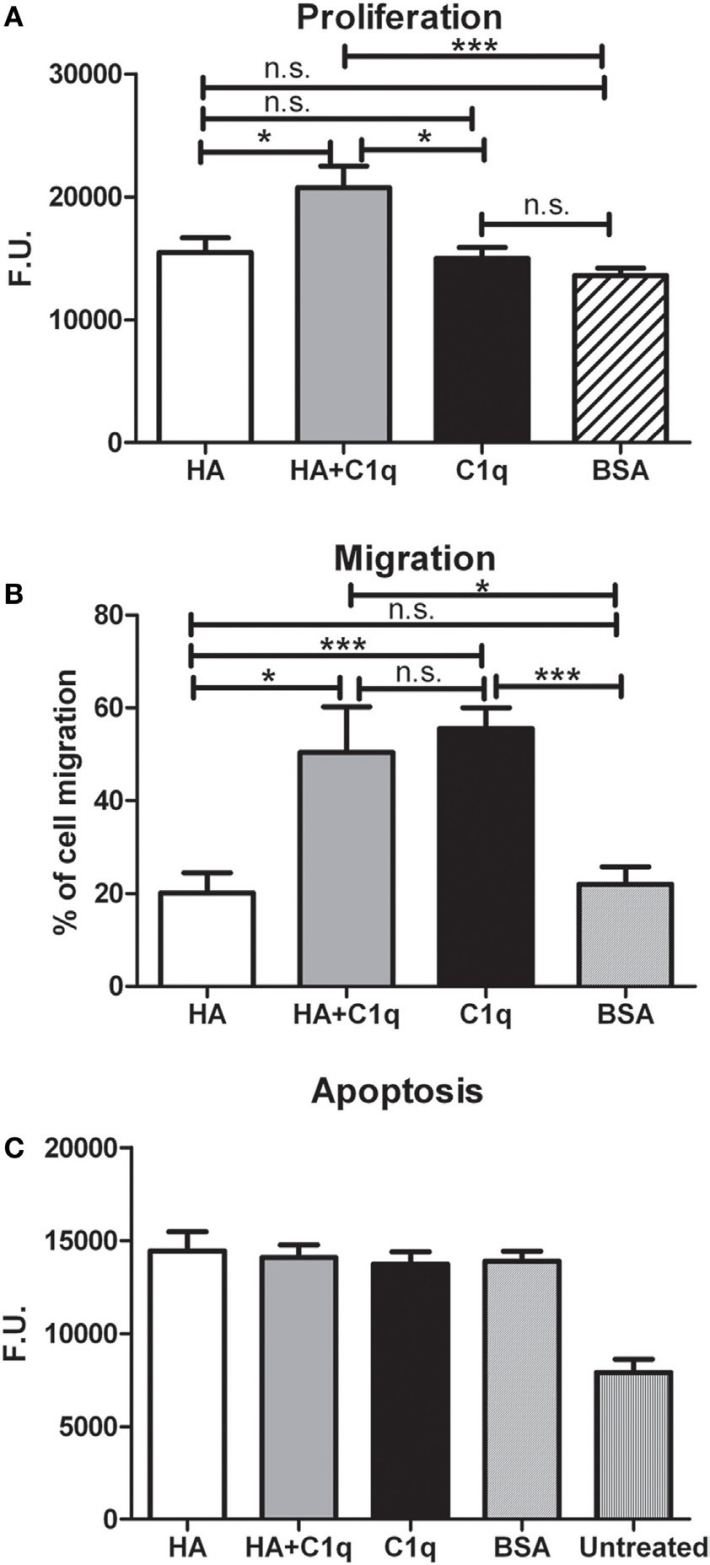
Effects of C1q on mesothelioma cells (MES) migration, proliferation, and apoptosis. **(A–C)** The experiments of cell migration, proliferation and apoptosis were performed as detailed in Section “Materials and Methods.” **(A)** MES proliferation. MES were seeded in wells precoated with hyaluronic acid (HA), C1q, HA-bound-C1q, or bovine serum albumin (BSA) and the number of proliferating cells was evaluated using the Click-iT^®^ EdU Microplate Assay. **(B)** FAST DiI-labeled MES were allowed to migrate through a trans-well system polycarbonate membranes coated on the lower side with HA, HA-bound-C1q, C1q, or BSA. **(C)** Serum-starved MES cells were seeded in the wells precoated with HA, C1q, HA-bound-C1q, or BSA and incubated with H_2_O_2_. Apoptotic cells were ascertained by CellEvent™ Caspase-3/7 (Life Technologies). Data from at least five independent experiments are presented as mean ± SEM.

We investigated whether C1q might contribute to tumor growth by stimulating the proliferation of MES. Thus, MES were seeded on to solid phase C1q, HA, or HA-bound-C1q and the number of proliferating cells were evaluated using the Click-iT^®^ EdU Microplate Assay. Our results indicated that HA-bound C1q was able to enhance the proliferation rate of MES (Figure 8A), compared to HA or C1q alone.

C1q-induced migration of labeled MES was examined by monitoring cell migration from the upper chamber through an insert coated with C1q, or HA-bound-C1q. C1q (60%) was found to be more effective than HA (~20%), in effecting migration (Figure 8B). In this assay, we did not observe a synergistic effect of the double matrix (HA-bound-C1q). In fact, the percentage of the cell migration due to HA + C1q was comparable to that observed with C1q alone.

**Figure 9:**
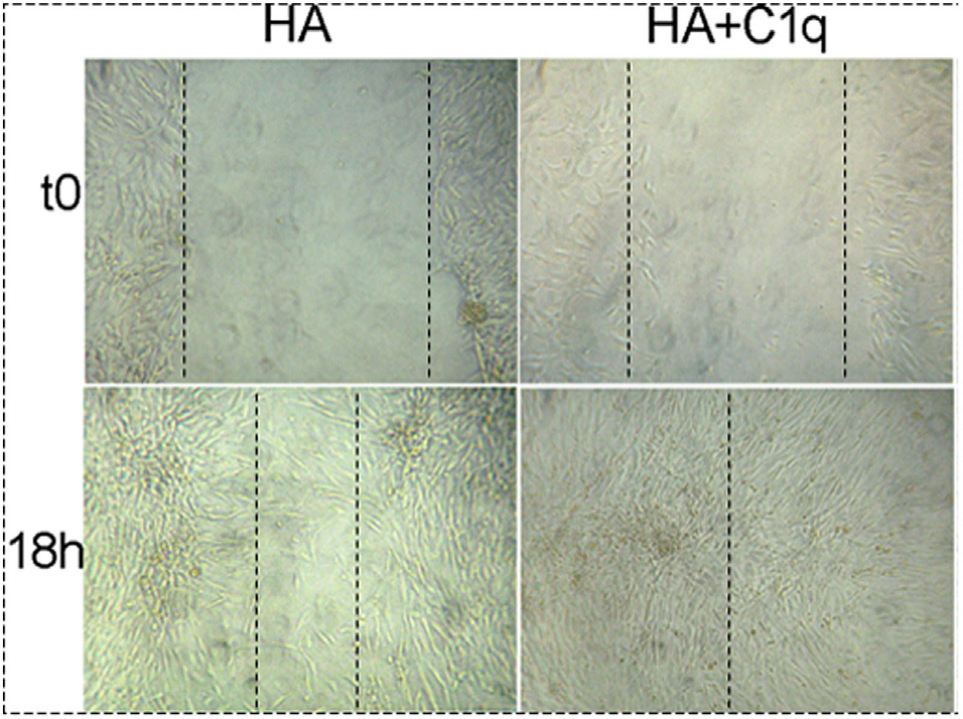
Effects of C1q on mesothelioma cells (MES) migration. For wound healing assays MES (2 × 10^5^) were seeded in HESF + 0.1% BSA medium in 24-well plate and grown to 90–95% confluence. A scratch was placed in the middle of the well with a sterile 200 µl pipette tip. Subsequently, hyaluronic acid (HA) (50 µg/ml) or HA + C1q (20 µg/ml) were added to the wells. The invasion was evaluated in three separate experiments performed independently. The results of a representative experiment are shown. Images were acquired by phase-contrast microscope, Leica original magnification: 200×.

The effect of C1q on the cell migration was also analyzed using a scratch wound healing assay, monitoring the mobilization of MES toward the scratched area for 18 h. As shown in Figure 9, MES stimulated by C1q started to enter the scratched area and migrated farther than cells exposed to HA after 18 h.

In order to investigate whether C1q was able to protect MES from apoptosis, serum-starved MES were allowed to adhere on to the wells, coated with HA or HA-bound-C1q, and then incubated with 500 µM H_2_O_2_ for 6 h. Subsequently, the activation of caspases 3 and 7 was detected using a fluorogenic substrate. As shown in the graph in Figure 8C, the fluorogenic units of the MES on HA treated with H_2_O_2_ was double, compared to the untreated cells. Surprisingly, MES adhering to C1q or HA-bound-C1q were not protected from apoptosis induced by oxidative stress (Figure 8C).

### C1q Enhances ERK1/2, SAPK/JNK, and p38 Phosphorylation in MES Cells

To further elucidate the mechanism of the cell activation, we analyzed the signaling pathways likely to be involved in tumor cell adhesion, migration and proliferation. We examined more specifically the activation of three members of the MAPK family, ERK1/2, SAPK/JNK, and P38 signaling molecules.

**Figure 10:**
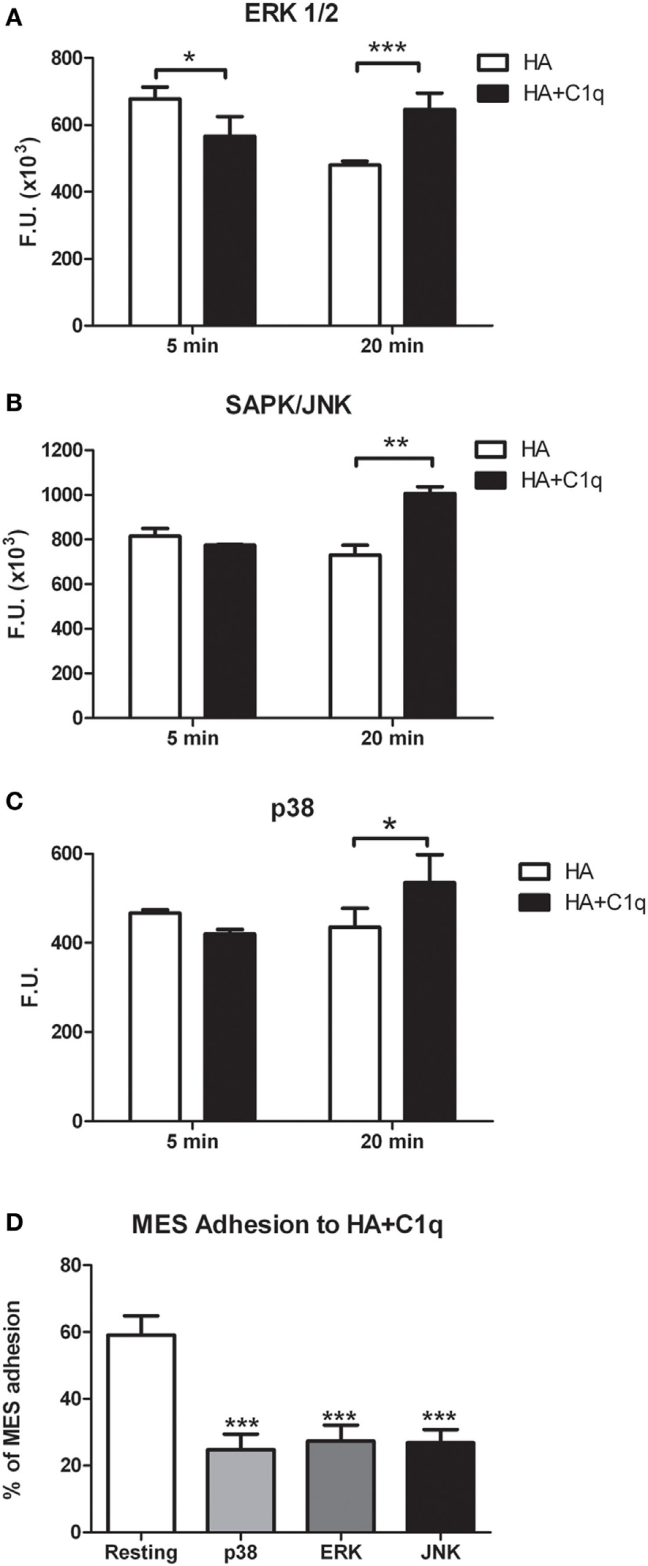
Phosphorylation of ERK1/2, SAPK/JUNK, and p38 in mesothelioma cells (MES). **(A–C)** MES were allowed to adhere to hyaluronic acid (HA) or HA-bound-C1q and the phosphorylation status of ERK1/2, SAPK/JUNK, and p38 was evaluated using total cell lysates with PathScan Antibody Array Kit (Cell Signaling), as described in the Section “Materials and Methods.” Data from at least three independent experiments are presented as mean ± SD. *p < 0.05, **p < 0.01, ***p < 0.001 (Student’s *t*-test). **(D)** Three different populations of MES were labeled with the fluorescent dye FAST DiI, preincubated for 30 min at 37°C with P38, ERK, or JNK inhibitors and then allowed to adhere to microtiter wells precoated with HA-bound-C1q. The data are expressed as mean of three independent experiments done in triplicates ± SEM. ***p < 0.001 vs. resting.

Serum-starved MES were allowed to adhere to wells coated with HA or HA-bound-C1q, for 5 or 20 min and the phosphorylation status of ERK1/2, SAPK/JNK, and P38 was evaluated by immunofluorescence using PathScan^®^ Intracellular Signaling Array Kit (Cell Signaling Technology). As shown in Figures 10A–C, binding of MES to HA-bound-C1q resulted in the activation of all the signaling molecules, which was clearly seen at 20 min post stimulation for all pathways and the signal was significantly higher in the cells adhering to C1q bound to HA than in cells adherent to HA only. All three MAPK inhibitors tested in the adhesion assay proved to be effective in reducing significantly the cell adhesion to C1q bound to HA (Figure 10D).

## Discussion

C1q is expressed in the stroma and vascular endothelium of a number of human malignant tumors, including adenocarcinomas of colon, lung, breast, and pancreas. In a murine model of melanoma, C1q was found to promote cancer cell adhesion, migration and proliferation (18). Thus, as a pro-tumorigenic soluble factor, C1q can promote tumor progression by facilitating cancer cell seeding and angiogenesis. Here, we investigated the presence and the role of C1q in MPM for a number of reasons. MPM is an aggressive neoplasm with a poor prognosis, because it is resistant to chemotherapy and radiotherapy. Therefore, studying the microenvironment of this tumor can help devise novel therapeutic strategies. In this study, we particularly focused our attention on the interaction between C1q and HA and its implication on adhesion and proliferation of MPM cells. Understanding this C1q-HA interaction is of great importance given a unique expression pattern of C1q in mesothelioma tissues and a great relevance of HA in the biology of the MPM.

IHC revealed that C1q was present in all the histological variants examined and it seemed primarily associated with monocytoid cells, indicating that these cells might be the main source of C1q locally. C1q was also diffusely present in the tumor stroma and associated with the cell membrane of tumor cells mainly of the epithelioid histotype. This pattern of expression is similar to that in other solid tumors (18) and is reminiscent of its distribution in human decidua (17). There were also areas in which C1q could be found associated with small vessels, raising the possibility that C1q might exhibit a proangiogenic activity in this context, similar to that in wound healing (23).

We first investigated the interaction of purified human C1q with HA since MPM is associated with a high level of production of HA (24). HA is highly expressed in MPM because it is responsible for the lubrication of the pleural membranes and is secreted by the mesothelial cells (25). Here, we demonstrated that HA is abundantly present in MPM tissues, confirming an earlier study (22). HA has previously been shown to promote proliferation and migration of MPM cells through its interaction with hyaluronan receptor (26). We have previously shown that C1q can interact with HA (17). Here, we showed that C1q can bind to HMW-HA, and as expected, this binding does not induce complement activation. The interaction between C1 and HA has been studied in the past mainly considering its anti-complement activity (27, 28). The interaction of C1q with synovial HA in rheumatoid arthritis has been previously reported (29); however, C1q binds synovial antibodies that are covalently coupled to HA. Heated and then lyophilized HA binds C1q (and a range of complement components) better that native HA, probably *via* polyanionic charges (30). A comparable binding activity was also observed for LMW-HA, whose local accumulation can be detected in the tumor microenvironment as a consequence of enhanced synthesis and turnover of HMW-HA. Since LMW-HA, but not HMW-HA, can stimulate a number of biological processes (31, 32), it is likely that C1q can interfere with several functions mediated by HA, such as angiogenesis and inflammation. To dissect the functional contribution of each chain within the heterotrimeric globular domain of C1q, we investigated the binding properties of the recombinant ghA, ghB, and ghC modules. Our results demonstrated that the ghA module was the best binder of HA.

The proadhesive properties of C1q that we demonstrated *in vitro* for murine melanoma cells (18) were also evident for MES. In this study, we made a significant step forward since we were able to test several populations of MES isolated directly from human patients. Here, we demonstrated that the effects mediated by C1q-bound-HA were different from that observed for HA or C1q alone in terms of the number of adhering cells as well as morphology indicating that the presence of C1q can considerably modify the tumor microenvironment. The proadhesion effect of C1q seems to be restricted to the cells isolated from primary tumors while Met5A cells, which are representative of non-tumor mesothelium, adhere better to HA, compared to the adhesion on C1q-bound-HA or C1q alone. These data indicate that C1q can exert differential effects depending on the cell types and probably expression of putative receptors. Among various C1q receptors gC1qR, also called HA-binding protein-1, is an interesting molecule for its ability to bind both the gC1q domain of C1q and HA. Furthermore, CD44, the main receptor for HA, has been described as a possible docking signaling molecule for the interaction with gC1q (33). The nature and consequences of interaction between these cellular receptors and C1q-bound-HA is currently under investigation. Another interesting observation is that the adhesion of MES to C1q is predominantly mediated *via* the ghC module. Since ghA is the preferential binder to HA, the gC1q domain can act as a bridging molecule for anchoring the tumor cells to the ECM. C1q-bound-HA was able to promote the growth and the migration of MES *in vitro* confirming our previous results (18), obtained with B16/F10 murine cells and C1q bound to fibronectin. The finding that C1q alone did not exert a significant proliferating effect on MES seen previously on melanoma cells may be explained by differential response of cells derived from various tumors to C1q. It is also important to emphasize that this study was carried out on primary cells, which were freshly isolated from patients.

The activation of three members of the MAPK family, ERK1/2, SAPK/JNK, and p38 in this study is also consistent with the previously reported study (17).

That C1q can act as a tumor promoting factor in MPM confirms our recent data (18). In addition, Winslow et al. have observed that the three chains of C1q were highly expressed in the stroma of breast cancers with poor prognosis (34). On the contrary, Hong et al. reported that C1q is involved in the regulation of cancer cell survival and progression sustaining the activation of the tumor suppressor *WW-domain containing oxidoreductase* (WOX1). C1q downregulation enhanced prostate hyperplasia and tumorigenesis because of the lack of WOX1 activation (35). Recently, Kaur et al. have reported that C1q, *via* its gC1q domain, induced apoptosis in an ovarian cancer cell line SKOV3 *via* TNF-α induced apoptosis pathway involving upregulation of Bax and Fas (36). These contrasting observations appear to suggest that the function of C1q in the biology of the tumor is complex and is strongly dependent on the microenvironment. Our hypothesis is that C1q can be locally produced by non-tumor cells and can interact differentially to the different ECM components present in the tumor microenvironment. The presence of C1q or C1 in soluble phase or bound to the ECM can provide different stimuli to the tumor cells present in the microenvironment.

In conclusion, C1q is abundantly present in mesothelioma tissue, interacts with HA, and interferes with adhesion, migration and proliferation of MES. The role of C1q is more complex than previously thought and is likely to be dependent on the tumor microenvironment. The availability of the recombinant globular domain of C1q may have implications for therapeutic approaches.

## Ethics Statement

This study was carried out in accordance with the recommendations of governmental guidelines and approved by the CEUR (Comitato Etico Unico Regionale, FVG, Italy; number 34/2016), with written informed consent from all subjects. All subjects gave written informed consent in accordance with the Declaration of Helsinki.

## Author Note

This work is dedicated to the memory of Bulla Gabriele and all mesothelioma patients.

## Author Contributions

Conception and design: RB, CA, and UK. Development of methodology: RV and LA. Acquisition of data: RV, VB, FZ, PG, and BB. Analysis and interpretation of data (e.g., statistical analysis, biostatistics, computational analysis): CT, MC, AM, and BB. Writing, review, and/or revision of the manuscript: RB, UK, CA, and FT. Study supervision: RB and MC.

## Conflict of Interest Statement

The authors declare that the research was conducted in the absence of any commercial or financial relationships that could be construed as a potential conflict of interest.

## Abbreviations

MPM, malignant pleural mesothelioma; MES, mesothelioma cells; ECM, extracellular matrix; HA, hyaluronic acid; ghA, ghB and ghC: globular head region of human C1q A, B and C chain, respectively; MBP, maltose-binding protein; HMW, high molecular weight; LMW, low molecular weight.
